# Daily Relations Between Stress and Electroencephalography-Assessed Sleep: A 15-Day Intensive Longitudinal Design With Ecological Momentary Assessments

**DOI:** 10.1093/abm/kaac017

**Published:** 2022-05-15

**Authors:** Yang Yap, Natasha Yan Chi Tung, Jorja Collins, Andrew Phillips, Bei Bei, Joshua F Wiley

**Affiliations:** School of Psychological Sciences and Turner Institute for Brain and Mental Health, Monash University, Melbourne, Australia; School of Psychological Sciences and Turner Institute for Brain and Mental Health, Monash University, Melbourne, Australia; Department of Nutrition, Dietetics and Food, Monash University, Melbourne, VIC, Australia; School of Psychological Sciences and Turner Institute for Brain and Mental Health, Monash University, Melbourne, Australia; School of Psychological Sciences and Turner Institute for Brain and Mental Health, Monash University, Melbourne, Australia; School of Psychological Sciences and Turner Institute for Brain and Mental Health, Monash University, Melbourne, Australia

**Keywords:** Stress, Sleep, EEG, EMA, Daily, International students

## Abstract

**Background:**

Recent studies have found bi-directional relations between stress and sleep. However, few studies have examined the daily associations between stress and electroencephalography (EEG) measured sleep.

**Purpose:**

This study examined the temporal associations between repeated ecological momentary assessments of stress and EEG-estimated sleep.

**Methods:**

Ninety-eight international or interstate undergraduate students (*M*_age_ = 20.54 ± 1.64, 76.5% female, 84.7% Asian) reported their stress levels four times daily at morning awakening, afternoon, evening, and pre-bedtime across 15 consecutive days (>4,000 total observations). Next-day stress was coded as an average of morning, afternoon, and evening stress. Z-Machine Insight+ recorded over 1,000 nights EEG total sleep time (TST), sleep onset latency, wake after sleep onset, sleep efficiency (SE), slow-wave sleep (SWS), and rapid eye movement (REM) sleep duration. Multilevel models, adjusted for covariates (i.e., sociodemographic, health factors, and daily covariates) and lagged outcomes, tested the daily within- and between-level stress-sleep associations.

**Results:**

After adjusting for covariates, within-person shorter TST (*b = −*0.11 [*−*0.21, *−*0.01], *p* = .04), lower SE (*b = −*0.02 [*−*0.03, 0.00], *p =* .04), less SWS (*b = −*0.38 [*−*0.66, *−*0.10], *p* = .008), and less REM sleep (*b = −*0.32 [*−*0.53, *−*0.10], *p* = .004) predicted higher next-day stress. Pre-bedtime stress did not predict same-night sleep. No significant results emerged at the between-person level.

**Conclusions:**

These findings demonstrate that poor or short sleep, measured by EEG, is predictive of higher next-day stress. Results for sleep architecture support the role of SWS and REM sleep in regulating the perception of stress. Given that only within-person effects were significant, these findings highlight the importance of examining night-to-night fluctuations in sleep affecting next-day stress and its impact on daytime functioning.

## Graphical Abstract

**Graphical Abstract:**
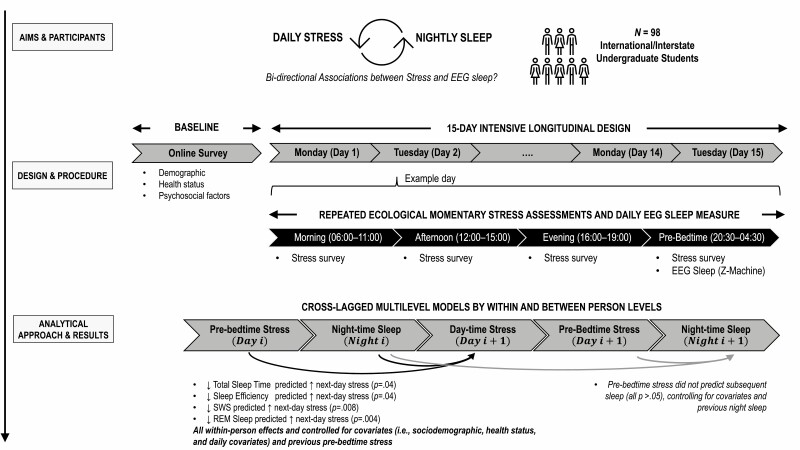

## Teaser

In a daily life study using gold-standard sleep measures, young adults reported higher stress the day after a night of less sleep than usual or worse quality sleep than usual. Experiencing more stress than usual during the day did not predict sleep that night.

## Introduction

Previous research has established cross-sectional associations between high stress and poor or short sleep [1], with both contributing to higher risks of poor health outcomes [2–4]. Recent studies have extended these findings by determining bi-directional or temporal associations between stress and sleep using daily sleep diary or actigraphy measures [5–10]. However, few studies to date have examined the relationship between stress and sleep using objective, electroencephalography (EEG) measures on a daily basis. Laboratory studies have shown restorative and emotional regulative benefits of slow-wave sleep (SWS) and rapid eye moment (REM) sleep [11–14]. We sought to determine whether laboratory findings translate to naturalistic conditions, and whether stress and sleep architecture bi-directionally influence each other on a daily basis in young adult undergraduate students. In recent years in developed countries over half of young adults pursue tertiary education, making this transitional period from high school to university a normative developmental period applying to most young adults. Within tertiary education students, those who move from a different state or country for the first time are particularly vulnerable as the normal transitional challenges may be compounded by relocation (e.g., adapt to living independently in new environments) and losing existing routine, community, and social support [15]. Furthermore, international students may experience additional acculturative stressors, such as language barriers and foreign educational systems [16, 17]. Although there is an increasing number of students who relocate for tertiary studies worldwide [18], which represents an at-risk subset of tertiary education students, no known studies have explicitly examined the day-to-day stress and sleep associations in this population.

Studies have examined daily stress-sleep relations, with those that explicitly tested the temporal order of the stress-sleep associations finding complex, bi-directional, and temporal associations [5–8]. Results differ across different aspects (e.g., duration, quality) and measurement of sleep (e.g., self-report, actigraphy) [5–8]. For instance, a 12-day study found that evenings with higher than usual stress levels predicted both shorter actigraphic and self-reported total sleep time (TST) that night, and that shorter actigraphic and self-reported TST predicted higher stress the next day [5]. Furthermore, worse than usual sleep quality (i.e., self-reported sleep onset latency [SOL], self-reported wake after sleep onset [WASO], and actigraphic and self-reported sleep efficiency [SE]) predicted higher next-day stress [5]. However, there is contrary evidence, showing that stress did not predict same-night self-reported TST, whereas self-reported TST and sleep quality predicted higher next-day stress [7]. Inconsistent findings may be due to differences in sleep measures. Self-reported sleep is susceptible to individuals’ mood state and perception about sleep; for example, those with insomnia and/or mood symptoms tend to underestimate TST and over-estimate wake [19]. Movement-based actigraphy on the other hand, may underestimate SOL compared to polysomnography (PSG) [20].

Neither self-reported nor actigraphic measures of sleep can accurately assess sleep architecture, which requires measurements of EEG. The sleep architecture consists of three non-rapid eye movement (NREM) sleep stages, that is, stage N1, N2, and N3, and REM sleep. A typical sleep cycle starts with entering the NREM sleep stages, followed by REM sleep. Each NREM sleep stage is progressively deeper and has unique brain waves, for example, rhythmic alpha waves in N1, sleep spindles and K-complexes in N2, and high-voltage, slow-wave-activity in N3. REM sleep has low-voltage, high-frequency brain wave activity [21]. Few studies have demonstrated effects of both experimentally induced and naturally occurring stress on subsequent EEG-assessed sleep. Individuals exposed to emotional stress showed significantly decreased TST and SE [22], as well as less REM sleep [22–24]. Similarly, individuals had significantly lower SE, but not TST, during high-stress periods compared with low-stress periods [24]. Reported findings for SWS are inconsistent, as individuals exposed to emotional stress or experiencing high-stress periods have showed an increase, decrease, or no change in SWS [22–25].

When people experience sleep loss, they tend to exhibit greater psychological stress and emotional reactivity [11, 26]. Evidence from neuroimaging studies suggests that SWS and REM sleep may play an emotional regulatory role, which may potentially explain these findings [13, 14]. For example, individuals who had a night of sleep deprivation reported higher anxiety levels the following morning compared to the previous night and to well-rested individuals; within well-rested individuals, longer SWS was associated with lower levels of next-day anxiety [14]. Another study showed that well-rested individuals with 8 hours of sleep opportunity had decreased amygdala and emotional reactivity toward affective images, compared with sleep-deprived individuals. Within well-rested individuals, low EEG gamma activity (a biomarker of adrenergic activity that plays a role in emotional regulation and amygdala activity) during REM sleep was associated with reductions in both amygdala activity and emotional reactivity towards the affective stimuli [13]. These findings show that SWS and REM sleep are associated with mood and emotional reactivity. Although perceived stress was not examined in these studies, given the strong link between stress and emotions, it is possible that SWS and REM sleep also may regulate one’s perception and appraisal of stress severity in addition to emotional responses. However, this notion remains untested, especially in daily settings.

The use of daily diary designs with repeated ecological momentary assessments (EMAs) allows daily variations in stress to be related to subsequent or previous sleep architecture. Furthermore, these designs allow ecological changes in stress or sleep to be studied in naturalistic settings, rather than relying on extreme manipulations used in experimental studies (e.g., sleep deprivation). Only one study to date examined the daily stress-sleep relations, using a 7-day daily diary design and EEG sleep measures [27]. The findings showed that stress did not predict any of the subsequent EEG sleep variables, and the EEG sleep measures did not significantly predict next-day stress.

The current study aimed to examine the bi-directional and temporal associations between stress and sleep across 15 days, using an intensive longitudinal design with repeated EMAs and a single-channel EEG sleep measure in young adults who are international or interstate undergraduate students. Specific hypotheses were: (a) higher pre-bedtime stress will predict subsequent shorter sleep duration (TST), worse sleep quality (i.e., longer SOL, higher WASO, and lower SE), and less SWS and REM sleep duration. (b) Shorter sleep duration, worse sleep quality, and less SWS and REM sleep will predict higher next-day stress.

## Methods

### Participants

**Fig. 1.**
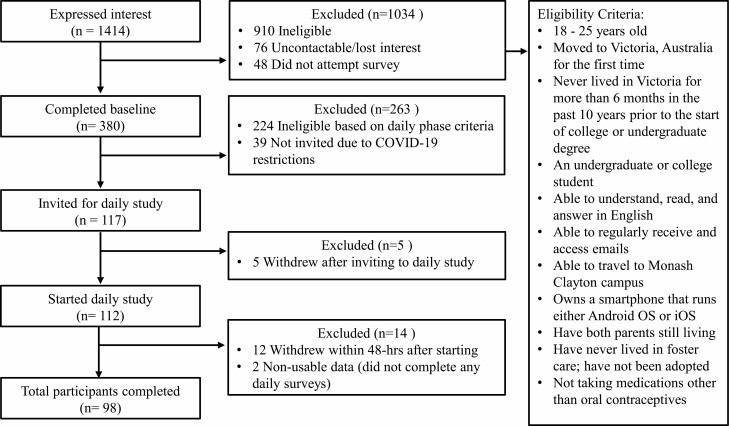
Summary of the Stress and Health Study recruitment process. The recruitment period was between February 2019 and June 2020.

The Stress and Health Study was conducted from February 2019 to June 2020 and recruited participants who had moved from a different state or country for undergraduate studies in Victoria, Australia. All participants were at any year in their college or undergraduate degree, and they moved to Victoria for the first time and had not previously spent more than 6 months in Victoria in the past 10 years before starting their college or undergraduate degree. Participants were recruited through the Monash Institute of Cognitive and Clinical Neurosciences Research Participation Platform, social media (e.g., Facebook), word-of-mouth, in-class presentations, and learning management systems (e.g., Moodle posts). Figure 1 summarizes the participant flow chart and eligibility criteria of the Stress and Health Study. A priori power analysis indicated that 68 participants, with the assumption of a 75% completion rate, would provide 80% power to detect small-to-medium effect sizes at the within-person level for stress and sleep. Additional participants were recruited to account for potential attrition, missing data, and other aims not related to the current paper.

### Design and Procedure

**Fig. 2.**
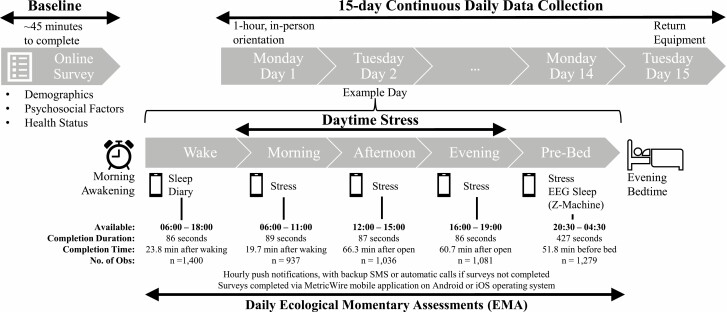
Diagram of the Stress and Health Study procedures. The top of the diagram shows the initial, baseline online survey phase after which eligible participants were invited to 15-day daily phase of the study, which always commenced on a Monday and completed on a Tuesday 2 weeks later. Daily measures were completed via the MetricWire mobile phone application and night-time EEG sleep via the Z-Machine Insight+. During the daily phase, participants completed five surveys per day via MetricWire (middle of diagram) assessing self-reported sleep via sleep diary and daytime and evening stress. Participants were provided training on how to complete the surveys and wear the Z-Machine Insight+ during the 1-hour, in-person orientation. Participants were instructed to clean their skin (with the provided alcohol wipes) and attach the disposable sensors (i.e., one-time use) approximately 30-minutes before their bedtime. Participants were instructed to only attach the cables to the EEG device when attempting to sleep. In addition to hands-on demonstration, participants also were provided with an instruction manual for reference at home. Surveys were available during a broad window with the median number of seconds to complete each survey shown in the figure as well as the median timing in minutes (min) of each survey, relative to wake, open of the available period, or bedtime. No. of Obs. is the number of observations available for each survey.

Monash University Human Research Ethics (project ID: 17281) approved all procedures, and all participants provided consent. This study used an intensive longitudinal design with repeated EMA for 15 consecutive days. This approach captures real-time variability of experiences in naturalistic settings, maximizing external validity and reducing memory and other biases related to conventional retrospective recall methods [28, 29]. Participants also served as their own control through the repeated assessments across days. These methods provide a rigorous test of directionality and temporal order between stress and sleep (e.g., examining pre-bedtime stress collected *during* pre*-*bedtime predicting subsequent sleep that night) [29]. Detailed procedures are in Fig. 2.

### Measures

#### Sleep

Daily objective estimates of sleep (i.e., TST, SOL, WASO, SE, SWS, and REM sleep) were measured in 30-second epochs using the Z-Machine Insight+, a portable, single-channel EEG sleep-monitoring device. There were three sensors: one sensor (signal) was placed behind each ear (i.e., differential mastoids A_1_ and A_2_), and one on the center of the neck below the hairline (ground). Raw EEG signals were automatically scored as sleep or wake through the Z-ALG13 (for more information on the Z-ALG algorithm and scoring methods, see original publication by Kaplan et al. [30]). The scored data were then processed by the Z-PLUS [31] algorithm to determine sleep stages as light (N1 and N2), SWS, or REM sleep. Previous studies showed that the Z-ALG13 has high sensitivity and specificity for determining sleep (95.5% and 92.5%, respectively), and the Z-PLUS has positive predictive values of 0.85 for light sleep, 0.83 for SWS, and 0.76 for REM sleep in normal, healthy sleepers compared to PSG consensus [30, 31]. Reported sensitivities of Z-PLUS are 0.83 for light sleep, 0.77 for SWS, and 0.74 for REM sleep [31]. Overall kappa agreement is 0.85 and 0.72 for Z-ALG13 and Z-PLUS, respectively [31]. Self-report sleep measures in our study were adapted from the Consensus Sleep Diary [32] and included bedtime, rise time, SOL, number of awakenings, and WASO.

#### Daily stress

Daily stress was measured using a self-reported adaptation from the Daily Inventory of Stressful Events scale [33, 34], completed four times each day (i.e., mornings, afternoons, evenings, and pre-bedtime). The number of surveys completed for each timepoint, alongside completion duration and time, are summarized in Fig. 1. In this study, we focused on a single item ranging from 0 (not at all stressful) to 10 (very stressful), that is, “Since the previous survey, how stressful has your day been?”. Stress values from the morning, afternoon, and evening surveys (see Fig. 2 for survey periods) were averaged by participant and day to create a composite of daily stress with ω _within_ = 0.635, ω _between_ = 0.98, indicating adequate and excellent reliability at the within and between levels, respectively. In analysis, daily stress was examined following sleep, thus named as next-day stress.

#### Covariates

Covariates were determined based on research demonstrating their associations with stress and sleep. Previous studies have shown several sociodemographic variables that are associated with stress or sleep. For example, young adults who are females (vs. males) [35, 36], non-White (vs. White) [37, 38], or reported lower subjective social status [39] had reported higher stress or poorer sleep. Thus, the sociodemographic variables included as between-person covariates in our model include: age (years) [40], sex (coded as male/female) [35, 36], race/ethnicity (coded as White/Asian/other) [37, 38], employment status (coded as working/not working) [41], student status (coded as international/interstate) [17], time spent in Victoria (years), English language acculturation (using the adapted Short Acculturation Scale for Hispanics to refer to participants’ native language instead of Spanish) [16, 42], and subjective social status [39, 43]. Furthermore, COVID-19 period (coded as pre [before Victoria lockdown 2020 March 8] vs. during) was included as a between-person covariate, given that recent study showed the impact of the pandemic on psychological well-being and sleep [44]. Several confounding health factors that can impact stress or sleep were also included. For example, higher body mass index (BMI), smoking, and alcohol consumption are associated with higher stress and poorer sleep in young adults [45–48]. Thus, BMI (kg/m^2^ from self-reported height and weight), smoking (coded current/former vs never), and alcohol risk (coded as abstainers/moderate/at-risk based on the National Institute on Alcohol Abuse and Alcoholism guidelines [49] using the first three items of the World Health Organization Alcohol Use Identification Test [50]) were included as between-person covariates. Daily covariates that may impact experiences of stress and sleep included day of the week, such that individuals have longer sleep and experience lower negative affect during weekends, and daily circadian misalignment (measured using Composite Phase Deviation) [51], as it is associated with affect and sleep architecture [52–54].

### Analytic Approach

Multilevel linear models were run in R (v.4.0.3), using restricted maximum likelihood and lme4 v1.1-13 to estimate the models, and lmerTest v2.0-33 to estimate degrees of freedom and *p*-values. Cohen’s *f*^2^ type effect size for all predictors was also calculated. These models, separated by sleep variable, tested the temporal order and bi-directional associations between stress and sleep at between (i.e., interindividual differences; the participants’ own average) and within-person levels (i.e., deviations from the individual’s own average across the 15 days), and included lagged outcomes to allow for a rigorous test of directionality. The first set of models tested daily pre-bedtime stress levels as predictors of sleep that night (TST, SOL, WASO, SE, SWS, and REM sleep), controlling for previous night sleep. The second set of models tested next-day stress levels as the outcome of previous night sleep, controlling for previous night pre-bedtime stress. The number of lagged variables to be included in these models was determined through stepwise addition (i.e., first to fourth order stress and sleep lags) and model comparisons using the Bayesian Information Criterion (BIC). All models showed that the first-order lag was the most appropriate model (i.e., lowest BIC value). Fixed effects included all covariates and between- and within-person predictors, whereas random effects included intercepts, lagged outcome variables, and within-person predictors. These models were also applied to self-reported sleep variables; these results are reported in Electronic Supplementary Material (Tables S2 and S3). Intraclass correlations (ICC; between-person level variance/total variance) for the stress and sleep variables reported in Table 1 showed that a high proportion of the variance is within-person (45%–74%), justifying the use of multilevel models.

**Table 1.** Descriptive Statistics for Demographic and Daily Variables (*N* = 98)

	*M* (SD)/*N* (%)	No. of Obs	ICC
Participant characteristics			
Age (years)	20.54 (1.64)	98	—
Time spent in Melbourne (years)	0.73 (0.94)	98	—
Body mass index (kg/m^2^)	21.94 (3.48)	98	—
Language acculturation	3.85 (1.02)	98	—
Subjective social status	5.52 (1.44)	98	—
Sex		98	
Male	20 (20.50)	—	—
Female	75 (76.50)	—	—
Others	3 (3.00)	—	—
Race/ethnicity		98	
Asian	83 (84.70)	—	—
White/European	9 (9.20)	—	—
Others	6 (6.10)	—	—
International student (vs. interstate)	90 (91.80)	98	—
Working (vs. not working)	22 (22.40)	98	—
Never smoked (vs. current/former)	94 (95.90)	98	—
Before COVID-19 period (vs. During)	72 (73.50)	98	—
Alcohol risk		98	
Abstainer	23 (23.50%)	—	—
Moderate	64 (65.30%)	—	—
At risk	11 (11.20%)	—	—
Daily study variables			
Stress levels			
Pre-bed	2.49 (1.63)	1,279	.38
Next-day	1.93 (1.49)	1,359	.50
Self-reported sleep			
Total sleep time (hr; range: 0.23–12)	7.44 (0.96)	1,379	.27
Sleep onset latency (min; range: 0–120)	25.83 (43.59)	1,394	.55
Wake after sleep onset (min; range: 0–180)	5.77 (7.63)	1,396	.29
Sleep efficiency (%; range: 40–100)	93.69 (7.81)	1,379	.48
EEG-estimate sleep			
Total sleep time (hr; range: 0.50–11.36)	6.23 (0.90)	1,086	.27
Sleep onset latency (min; range: 0.50–149.26)	22.74 (11.53)	1,272	.26
Wake after sleep onset (min; range: 4.50–259.50)	48.69 (22.51)	1,086	.37
Sleep efficiency (%; range: 14.25–97.28)	83.82 (5.78)	1,086	.32
SWS (hr; range: 0–2.89)	1.43 (0.30)	1,086	.31
REM sleep (hr; range: 0–4.00)	1.64 (0.44)	1,086	.40

EEG-estimated and self-reported sleep efficiency, sleep onset latency, and wake after sleep onset presented are raw values.

*ICC* intraclass correlations, the proportion of total variance between people; *No. of Obs* number of observations; *EEG* electroencephalogram; *SWS* slow wave sleep; *REM* rapid eye movement sleep.

All dependent variables and model diagnostics were checked for relevant assumption violations. Due to skewness, SOL and WASO were square-root transformed and winsorized and SE was winsorized (top and bottom 0.5%). For nights with EEG sleep recordings that were identified as sensor errors or battery issues after the first sleep epoch, TST, WASO, SE, SWS, and REM were set as missing; SOL was retained given that it occurred before sleep. Model convergence failure was addressed using the Nelder-Mead algorithm and tightening tolerance values. If the singularity persisted, the random effect variable with the lowest variance was dropped from the model.

## Results

### Descriptive

Figure 1 summarizes the participant flow chart and eligibility criteria of the current study. From 117 participants who were invited to the daily study, 5 withdrew prior to starting, and 12 withdrew within 48 hr after starting the daily study. Two participants were excluded due to not completing any daily surveys. The final sample consisted of 98 participants (*M*_age_ = 20.54, *SD* = 1.64 years). Most participants were female (76%), of Asian descent (84%), and were international students (91%) who had spent less than a year in Melbourne. Only 5% of the sample is currently taking oral contraceptives, and the others were currently not taking any medications. Most participants had a BMI within the healthy adult range (18.5–24.9 kg/m^2^), were moderate drinkers (65%), and had never smoked (95%). Table 1 shows the number of observations, descriptive statistics for demographic and daily variables, alongside ICCs for all daily variables.

On average, participants’ pre-bedtime and next-day stress level throughout the study period were 2.49 ± 1.63 and 1.93 ± 1.49 (*M* ± SD; possible range 0–10), respectively, representing normative stress levels comparable to daily stress levels reported in other studies including healthy undergraduate students in Australia [5] and adults [9]. Participants reported the highest number of work or university-related stressors (13.94%), followed by the argument (6.24%), health (5.13%), home (2.68%), others (1.89%), finance (1.73%), relationship (1.42%), and discrimination (0.55%). Participants’ average self-reported TST was 7.44 ± 0.96 hr, within the recommended sleep duration for adults, and the average self-reported SE was 94 ± 8%, indicative of good sleep quality [55, 56]. However, EEG-estimated average TST was 6.23 ± 0.90 hr, below the recommended sleep duration for adults, and average SE was 83 ± 6%. The proportion of SWS and REM sleep were typical of the healthy young adult population without sleep complaints [57]. On average, participants completed 73% of all possible stress surveys (i.e., across morning, afternoon, evening, and pre-bedtime). Across all nights, 74% of the EEG TST, WASO, SE, SWS, and REM, as well as 86% SOL, were usable for analysis.

There were no significant differences in stress levels or EEG sleep variables between international and interstate students (all *p* ≥ .37). Comparing pre and during the COVID-19 period, there were no significant differences in stress levels, or in self-reported or EEG-estimated TST and SE. However, participants during the COVID-19 period had significantly shorter overall SWS (1.22 ± 0.28 hr) compared to individuals during pre-COVID-19 (1.50 ± 0.28 hr), *p* < .001. These results are reported in Electronic Supplementary Material (Table S1).

### Pre-bedtime Stress Predicting EEG-Estimated Sleep

Table 2 shows the unadjusted and adjusted cross-lagged multilevel models of pre-bedtime stress predicting EEG-estimated sleep, showing the between-person and within-person effects. For the unadjusted models, between-person effects showed that individuals with generally higher pre-bedtime stress had longer SOL (*b =* 0.17, 95% CI [0.02, 0.32], *p* =.03), lower SE (*b=* -0.87, 95% CI [-1.62, -0.12], *p* =.03), and less REM sleep (*b =* -4.51, [-8.12, -0.91], *p* =.02), adjusting for previous night sleep. However, after adjusting for covariates, pre-bedtime stress did not significantly predict sleep at either the between- or within-person levels. Similarly, the post-hoc exploratory analyses also showed that pre-bedtime stress did not predict self-reported sleep at either the between- or within-person levels (Supplementary Table S2). For covariates, the most reliable associations were daily circadian misalignment and day of week significantly associated with sleep (see Supplementary Table S4 for significance and specific directions for each sleep variable).

**Table 2.** Cross-Lagged Multilevel Model Testing Pre-bedtime Stress as Predictor of EEG-Estimated Sleep

	Between-person unadjusted	Within-person unadjusted	Between-person adjusted	Within-person adjusted
TST (min)	−1.58 [−8.67 to 5.51] *f*^2^ < 0.01	−1.44 [−4.24 to 1.36] *f*^2^ < 0.01	3.36 [−5.09 to 11.81] *f*^2^ < 0.01	−0.25 [−3.14 to 2.64] *f*^2^ < 0.01
SOL (√min)	0.17* [0.02 to 0.32] *f*^2^ = 0.02	−0.03 [−0.08 to 0.02] *f*^2^ < 0.01	0.16 [−0.04 to 0.35] *f*^2^ < 0.01	−0.03 [−0.09 to 0.04] *f*^2^ < 0.01
WASO (√min)	0.20 [0.00 to 0.39] *f*^2^ = 0.02	−0.005 [−0.07 to 0.06] *f*^2^ < 0.01	0.09 [−0.15 to 0.33] *f*^2^ < 0.01	0.005 [−0.06 to 0.07] *f*^2^ < 0.01
SE (%)	−0.87* [−1.62 to −0.12] *f*^2^ = 0.03	0.08 [−0.18 to 0.35] *f*^2^ < 0.01	−0.30 [−1.20 to 0.61] *f*^2^ < 0.01	0.09 [−0.17 to 0.36] *f*^2^ < 0.01
SWS (min)	−0.98 [−3.36 to 1.40] *f*^2^< 0.01	0.35 [−0.51 to 1.22] *f*^2^ < 0.01	−0.71 [−3.33 to 1.90] *f*^2^ < 0.01	0.44 [−0.48 to 1.36] *f*^2^ < 0.01
REM (min)	−4.51* [−8.12 to −0.91] *f*^2^ = 0.03	−0.84 [−1.99 to 0.30] *f*^2^ < 0.01	−1.92 [−6.20 to 2.36] *f*^2^ < 0.01	−0.76 [−1.94 to 0.42] *f*^2^ < 0.01

Results are unstandardized regression coefficients, asterisks to indicate significance, [95% confidence intervals], Cohen’s *f*^2^. Adjusted models included baseline and daily covariates: age; sex; race/ethnicity; body mass index; employment status; English language acculturation; subjective social status; time spent in Melbourne; COVID-19 period; student status; smoking status; alcohol consumption; day of week; composite phase deviation.

*TST* total sleep time; *SOL* sleep onset latency (square-root transformed); *WASO* wake after sleep onset (square-root transformed), *SE* sleep efficiency (winsorized); *SWS* slow wave sleep; *REM* rapid eye movement.

*p < .05.

### EEG-estimated Sleep Predicting Next-day Stress

Adjusted and unadjusted models of EEG-estimated sleep predicting next-day stress are summarized in Table 3. In the unadjusted models, between-person effects showed that longer SOL (*b =* 0.35, 95% CI [0.09 to 0.61], *p* = .009), lower SE (*b =* −0.07, 95% CI [−0.13 to −0.02], *p* = .02), and less REM sleep (*b* = −0.84, 95% CI [−1.55 to −0.16], *p* = .02) predicted higher next-day stress, adjusting for previous pre-bedtime stress. Within-person effects showed similar trends, with longer SOL (*b =* 0.06, 95% CI [0.01 to 0.11], *p* =.02), lower SE (*b =* −0.01, 95% CI [−0.02 to 0.00], *p =*.047), less REM sleep (*b* = −0.21, 95% CI [−0.39, −0.03], *p* = .02), and less SWS (*b* = −0.36, 95% CI [−0.59, −0.13], *p* = .002) predicting higher next-day stress.

**Table 3.** Cross-lagged Multilevel Model Testing EEG-Estimated Sleep as a Predictor of Next-Day Stress

	Between-person unadjusted	Within-person unadjusted	Between-person adjusted	Within-person adjusted
TST (h)	−0.08 [−0.45 to 0.29] *f*^2^ <.01	−0.06 [−0.15 to 0.03] *f*^2^ = 0.04	0.22 [−0.21 to 0.65] *f*^2^ = 0.01	−0.11* [−0.21 to −0.01] *f*^2^ = 0.03
SOL (√min)	0.35** [0.09 to 0.61] *f*^2^ = 0.04	0.06* [0.01 to 0.11] *f*^2^ < .01	0.32 [0.00 to 0.64] *f*^2^ = 0.04	0.05 [−0.02 to 0.11] *f*^2^ < .01
WASO (√min)	0.19 [−0.02 to 0.40] *f*^2^ = 0.02	−0.01 [−0.06 to 0.04] *f*^2^ < .01	0.19 [−0.01 to 0.40] *f*^2^ = 0.02	−0.02 [−0.07 to 0.03] *f*^2^ < .01
SE (%)	−0.07* [−0.13 to −0.02] *f*^2^ = 0.04	−0.01* [−0.02 to 0.00] *f*^2^ < .01	−0.03[−0.10 to 0.04] *f*^2^ = 0.01	−0.02* [-0.03 to 0.00] *f*^2^ = 0.01
SWS (hr)	−0.80 [−1.90 to 0.30] *f*^2^ = 0.01	−0.36** [−0.59 to −0.13] *f*^2^ = 0.01	−0.92 [−2.38 to 0.54] *f*^2^= 0.01	−0.38** [−0.66 to −0.10] *f*^2^ = 0.02
REM (hr)	−0.84* [−1.55 to −0.16] *f*^2^ = 0.04	−0.21* [−0.39 to −0.03] *f*^2^ = 0.01	−0.44 [−1.28 to 0.40] *f*^2^ = 0.01	−0.32** [−0.53, −0.10] *f*^2^ = 0.02

Results are unstandardized regression coefficients, asterisks to indicate significance, [95% confidence intervals], Cohen’s *f*^2^. Adjusted models included baseline and daily covariates: age; sex; race/ethnicity; body mass index; employment status; English language acculturation; subjective social status; time spent in Melbourne; COVID-19 period; student status; smoking status; alcohol consumption; day of week; composite phase deviation.

* *p* < .05, **p < .01.

Adjusting for covariates, within-person effects showed that when individuals had shorter TST (*b =* −0.11, 95% CI [−0.21 to −0.01], *p* = .04), lower SE (*b =* −0.02, 95% CI [−0.03 to 0.00], *p =* .04), less SWS (*b =* −0.38, 95% CI [−0.66 to −0.10], *p* = .008), and less REM sleep (*b =* −0.32, 95% CI [−0.53 to −0.10], *p* = .004) relative to their average levels, they had higher stress the following day. No significant associations were found for between-person sleep and next-day stress. For self-reported sleep, shorter within-person TST predicted higher next-day stress (*p* = .041); no other significant results emerged (Supplementary Table S3). For covariates, the most reliable association was sex significantly associated with next-day stress (see Supplementary Table S5 for specific directions).

## Discussion

This study examined the temporal relations between daily stress and EEG-measured sleep across a 15-day intensive longitudinal design with repeated EMA, extending the stress-sleep literature that is primarily based on cross-sectional evidence. All models controlled for lagged outcomes to provide a rigorous test of directionality. The results showed that pre-bedtime stress did not predict any of the subsequent EEG-sleep variables after accounting for covariates. However, compared to one’s own mean, nights with shorter TST, lower SE, less REM sleep, and less SWS predicted higher next-day stress, even after adjusting for covariates. These findings indicate a stronger unidirectional effect from sleep to next-day stress, compared to pre-sleep stress on sleep. Furthermore, after controlling for covariates, stress and EEG-sleep were not significantly associated on the between-person level, underlining the importance of considering day-to-day fluctuations in sleep.

### Pre-bedtime Stress Predicting Same-night Sleep

Findings from the unadjusted models, with lagged outcomes included, showed that individuals with generally higher pre-bedtime stress (i.e., between-person effects) had significantly longer SOL, lower SE, and less REM sleep. These findings are consistent with previous studies where individuals exposed to experimentally-manipulated stressors (e.g., emotional stress from a failure experience) or during high-stress periods had significantly lower EEG-estimated SE or less REM sleep compared with neutral groups or during low-stress periods [22–24]. However, these findings became non-significant when adjusted for baseline (e.g., demographics and health factors) and daily (e.g., day of week; circadian misalignment) covariates. These adjusted findings are similar to those of a recent 7-day study showing no association between daily stress and EEG sleep adjusting for day of week, gender, and age [27].

The nonsignificant findings could be due to our sample not being powered to detect small effect sizes or that in young adults, naturalistic levels of stress do not predict subsequent sleep. This direction is also observed in other daily studies that showed a more consistent direction of sleep predicting stress, rather than stress predicting sleep [8, 10]. It is possible that major stressful events (e.g., after traumatic events; severed relationships) may have a larger and more consistent impact on sleep compared to daily stress [25]. Although our sample consisted of international and interstate students, their average daily stress levels were similar to other undergraduate students and international students in Australia [5], suggesting that despite additional demands due to moving and entering another culture, these students have normative daily stress levels. There also may be differences between perceived stress and physiological stress responses (e.g., cortisol). For example, in a sample of nurses and physicians, increased cortisol responses were associated with occurrences of stressful events (e.g., medical emergency; routine care), but over 70% of these responses occurred without individuals perceiving the events as stressful [58]. Future studies should incorporate physiological markers, such as collecting daily cortisol sample or other biomarkers (e.g., skin conductance, heart-rate variability) in addition to perceived stress to understand interactions between psychological and physiological stress on daily sleep.

### EEG-Estimated Sleep Predicting Next-day Stress

After adjusting covariates, EEG sleep variables did not significantly predict next-day stress on the between-person level. On the within-person level, when individuals had a shorter than their own usual TST, SWS, or REM sleep, or lower than their usual SE, they experienced higher stress levels the following day even after adjusting for covariates. This could be due to the large number of covariates at the between-person level (i.e., 12 between-person covariates vs. 2 daily covariates). Furthermore, these results were adjusted for pre-sleep stress levels of the previous night, so the associations cannot be attributed to sustained or carry-over stress from the previous day. These results are in line with previous daily studies using actigraphic and self-reported sleep measures that linked shorter and poorer sleep with higher stress [5, 6]. Our findings contrast with the null findings from the previous study examining nightly EEG sleep and daily stress [27]. This could be due to differences in assessing stress levels (e.g., retrospective recall assessing stress once per day in the mornings vs EMA in this study), analytical approach (i.e., we included lagged outcomes and influential covariates), or population as our sample was younger and diverse racially versus middle-aged and predominantly White. Our findings also add to the growing evidence consistent with emotional regulatory benefits of SWS and REM sleep [13, 14].

The observed stress-sleep relationships could be explained by previous studies showing the impact of short and poor sleep on the emotional regulatory system by potentiating emotional responses to stressor intensity [26, 59]. This interpretation is supported by our findings that less SWS and REM sleep predicted higher next-day stress, in accord with studies that have uncovered the functional role of SWS and REM sleep in mood and emotional regulation [11, 13, 14]. For example, a recent study suggested the anxiolytic benefits of SWS, as longer SWS was associated with higher medial pre-frontal cortex activity (important for emotional regulation) and lower self-reported anxiety levels [14]. Furthermore, neurobiological frameworks have indicated the functional benefits of REM sleep in optimal emotional regulation [11]. Although emotional reactivity towards stressors were not tested in this study, given the robust link between stress and emotions, it is possible that short and poor sleep can impact one’s regulation of stress perception. In other words, it is possible that on nights when individuals had less SWS or REM sleep, they may have been deprived of the usual regulatory benefits, leading to a stronger reactivity to next-day stressors and thus perceiving them as more intense. Nonetheless, future studies are needed to further explore and clarify whether SWS and REM sleep predicts next-day emotional reactivity (e.g., increased negative affect and dampened positive affect) toward daily stressors on a daily, naturalistic basis.

### Strengths and Limitations

This study is one of the first to examine the daily associations between stress and sleep in an understudied population of young adults who relocated from a different state or country for undergraduate studies. Our rigorous study design included repeated EMAs to measure perceived stress levels multiple times across the day, EEG-derived estimates of sleep, and a 15-day intensive longitudinal design. These methods addressed key limitations of previous studies, such as recall biases, under- and overestimations of sleep using actigraphic and self-report measures, and the reliance of single time-point or cross-sectional evidence. This study explored the ecology of nightly variations in EEG-estimated sleep parameters and architecture in relation to next-day stress, extending the findings from sleep-manipulation studies conducted in laboratory settings. Furthermore, our rigorous analytical methods allowed for a robust test of temporal directionality by including both within- and between-person predictors and lagged outcomes in all models. The confidence in our findings and precision estimation are further strengthened by including important covariates, such as daily circadian misalignment.

Nonetheless, several limitations should be acknowledged. First, participants were young adults and relatively healthy, thus the findings cannot be generalized to other age groups (e.g., children, older adults) or populations with clinical conditions (e.g., individuals with insomnia or mood disorders). Future studies are needed to confirm these results in clinical populations and of different age groups, which may show and may show stronger associations and larger effect sizes. Second, the missing data in stress surveys and EEG-sleep measures may have influenced the results. For example, it is possible that participants may not wear the EEG device during nights with more stressful events, e.g., working on an assignment until late at night and going to bed immediately after, although we found no significant differences in pre-bedtime stress between nights with missing vs non-missing EEG sleep data. Relatedly, within-person reliability for next-day stress was on the low end of acceptable, which may explain some non-significant findings. It is also important to acknowledge that the current study only used a single-channel EEG to estimate the sleep parameters and architecture, which is still less accurate than the gold-standard PSG sleep measure. There also may be first-night effects using the Z-Machine Insight+, as with every other in-person PSG study, such that individual’s sleep on the first night may be impacted by wearing the device [60], although this is mitigated by the within-person levels of analyses in our study. It is worthwhile noting that we did not control for multiple comparisons, which may increase our chances for making Type 1 error. Given that this is one of the first studies examining daily EEG sleep in naturalistic settings, we think it is important to examine the multi-components of EEG sleep (i.e., parameters and architectures) and their associations with daily stress. Nonetheless, future studies are needed to replicate these findings. Finally, although every effort was made to robustly test the directionality of results through time-lagged predictions and covariates, our findings may still be influenced by unexplored confounds. Future studies involving experimentally-manipulated sleep in naturalistic settings (i.e., at home and, rather than severe sleep deprivation, manipulating sleep by minutes or hours) or populations with greater sleep disruption (e.g., shift workers) could provide evidence for whether there are causal effects of sleep on next-day stress in an ecologically valid context.

### Conclusion and Implications

Our findings showed that young adult perceived higher stress the next day after nights with shorter than usual EEG-measured TST, REM sleep, SWS, and poorer than usual sleep quality. However, higher pre-bedtime stress was not associated with same-night sleep over and above the effects of covariates. There are several implications, particularly for the educational sector and tertiary educational institutions as they relate to international students’ daily well-being. First, all significant associations were within-person effects, highlighting the importance of day-to-day fluctuation of sleep on each individual’s next-day experiences. The lack of robust between-person association between sleep and stress suggests that simply identifying poor sleepers may not be sufficient. The effect sizes are small (e.g., 8% higher SE [based on an approximate 30-minute increase in SOL and WASO and decrease in TST in the current sample] predict 0.16 higher stress the following day, with *f*^2^ = 0.01), which could be attributable to the relatively healthy sample in this study and the sleep measurement scale (e.g., SE percentage from 0 to 100). Although it is unclear if these within-person effects translate to clinical changes in this relatively healthy sample, these results show the ecology of the associations between stress and sleep in daily life. Nonetheless, having poor or short nightly sleep and high daily stress can cumulatively impact health [2–4], suggesting that behavioral sleep strategies that are applicable in everyday settings for managing the fluctuating nature of nightly sleep, especially on nights of particularly short or poor sleep are needed. Finally, this study identified sleep architecture (especially REM and SWS) as an aspect of sleep that is relevant to individuals’ experiences of stress. With the advancement of portable and consumer EEG sleep-monitoring devices, the incorporation of sleep architecture measures (e.g., SWS; REM) in addition to sleep quantity and continuity metrics (e.g., TST, SE) could be a fruitful area for future research to better understand sleep and its function in everyday life.

## Supplementary Material

kaac017_suppl_Supplementary_Material
