# Supplementary material for: Daily Relations Between Stress and Electroencephalography-Assessed Sleep: A 15-Day Intensive Longitudinal Design With Ecological Momentary Assessments
Source: Ann Behav Med. 2022 May 15;56(11):1144–56. doi: 10.1093/abm/kaac017 (PMC9635997; doi:10.1093/abm/kaac017)
Supplement: kaac017_suppl_Supplementary_Material [file kaac017_suppl_supplementary_material.docx]

Electronic Supplementary Material

| Table S1. *Descriptive Statistics for Demographic and Daily Variables by COVID-19 Period* | | | | |  |
| --- | --- | --- | --- | --- | --- |
| **Participant Characteristics** | Pre-COVID Period  *M* (SD) | During COVID Period  *M* (SD) | | *p*-value | |
| **Daily Study Variables** |  |  |  | |  |
| Stress levels |  |  |  | |  |
| Pre-Bed | 2.40 (1.60) | 2.76 (1.71) | .34 | |  |
| Next-Day | 1.85 (1.38) | 2.15 (1.77) | .37 | |  |
| Self-reported sleep |  |  |  | |  |
| Total sleep time (h) | 7.40 (1.01) | 7.56 (0.82) | .46 | |  |
| Sleep onset latency (min) | 25.79 (49.31) | 25.94 (21.63) | .99 | |  |
| Wake after sleep onset (min) | 5.05 (5.46) | 7.76 (11.64) | .12 | |  |
| Sleep efficiency (%) | 93.88 (8.65) | 93.16 (4.91) | .69 | |  |
| EEG-estimate sleep |  |  |  | |  |
| Total sleep time (h) | 6.21 (0.96) | 6.30 (0.71) | .64 | |  |
| Sleep onset latency (min) | 23.19 (11.52) | 21.51 (11.70) | .53 | |  |
| Wake after sleep onset (min) | 49.24 (24.75) | 47.18 (15.12) | .69 | |  |
| Sleep efficiency (%) | 83.62 (6.23) | 84.37 (4.35) | . 58 | |  |
| SWS (h) | 1.50 (0.28) | 1.22 (0.28) | <.001 | |  |
| REM sleep (h) | 1.62 (0.45) | 1.69 (0.43) | .51 | |  |

*Note.* EEG = Electroencephalogram. SWS = Slow Wave Sleep. REM = Rapid Eye Movement Sleep. EEG-estimated and self-reported sleep efficiency, sleep onset latency, and wake after sleep onset presented are raw values. Participants’ values were first averaged and *p*-values were based on the comparison between pre- and during COVID period using Independent-samples t-tests.

| Table S2. *Cross-lagged Multilevel Model Testing Pre-Bedtime Stress as Predictor of Self-Reported Sleep* | | | | |
| --- | --- | --- | --- | --- |
|  | **Between-Person**  **Unadjusted** | **Within-Person**  **Unadjusted** | **Between-Person**  **Adjusted** | **Within-Person**  **Adjusted** |
| **TST**  **(min)** | -1.66  [-9.86, 6.55]  *f*^2^<0.01 | -1.44  [-4.32, 1.45]  *f*^2^=0.02 | -1.55  [-11.41, 8.30] *f*^2^<0.01 | -0.77  [-3.51, 1.97]  *f*^2^<.01 |
| **SOL**  $\boldsymbol{(}\sqrt{\text{min}}$**)** | 0.09  [-0.21, 0.40]  *f*^2^<0.01 | -0.04  [-0.10, 0.02]  *f*^2^<0.01 | 0.17  [ -0.18, 0.51]  *f*^2^=0.01 | -0.04  [-0.10, 0.02]  *f*^2^<0.01 |
|  |  |  |  |  |
| **WASO**  $\boldsymbol{(}\sqrt{\text{min}}$**)** | -0.01  [-0.17, 0.14] *f*^2^<0.01 | 0.01  [-0.06 0.07]  *f*^2^=0.06 | 0.01  [-0.16, 0.19]  *f*^2^<0.01 | 0.01  [-0.04, 0.07]  *f*^2^<0.01 |
|  |  |  |  |  |
| **SE**  **(%)** | -0.33  [-1.69, 1.04]  *f*^2^<0.01 | 0.10  [-0.20, 0.40]  *f*^2^<0.01 | -0.62  [-2.17, 0.94]  *f*^2^<0.01 | 0.12  [-0.20, 0.44] *f*^2^<0.01 |
|  |  |  |  |  |

*Note*. Results are unstandardized regression coefficients, asterisks to indicate significance, [95% confidence intervals], Cohen’s *f*^2^.TST = Total Sleep Time; SOL = Sleep Onset Latency

(square-root transformed); WASO = Wake After Sleep Onset (square-root transformed), SE = Sleep Efficiency (winzorised). Adjusted models included baseline and daily covariates: age; sex; race/ethnicity; body mass index; employment status; English language acculturation; subjective social status; time spent in Melbourne; COVID-19 period; student status; smoking status; alcohol consumption; day of week; composite phase deviation.

| Table S3. *Cross-lagged Multilevel Model Testing Self-Reported Sleep as Predictor of Next-Day Stress* | | | | |
| --- | --- | --- | --- | --- |
|  | **Between-Person**  **Unadjusted** | **Within-Person**  **Unadjusted** | **Between-Person**  **Adjusted** | **Within-Person**  **Adjusted** |
| **TST**  **(h)** | 0.04  [-0.26, 0.34]  *f*^2^<0.01 | -0.04  [-0.10, 0.03]  *f*^2^=0.02 | -0.05  [-0.38, 0.29]  *f*^2^<0.01 | -0.07*  [-0.13, 0.00]  *f*^2^<0.01 |
|  |  |  |  |  |
| **SOL**  $\boldsymbol{(}\sqrt{\text{min}}$**)** | 0.03  [-0.11, 0.16]  *f*^2^<0.01 | 0.02  [-0.03, 0.07]  *f*^2^=0.03 | 0.10  [-0.05, 0.25]  *f*^2^=0.01 | 0.02  [-0.03, 0.06]  *f*^2^<0.01 |
|  |  |  |  |  |
| **WASO**  $\boldsymbol{(}\sqrt{\text{min}}$**)** | 0.10  [-0.17, 0.37]  *f*^2^<0.01 | 0.003  [-0.06, 0.07]  *f*^2^=0.04 | 0.15  [-0.18, 0.48]  *f*^2^=0.01 | 0.004  [-0.06, 0.07]  *f*^2^=0.03 |
|  |  |  |  |  |
| **SE**  **(%)** | -0.01  [-0.04, 0.02]  *f*^2^<0.01 | 0.004  [-0.01, 0.00]  *f*^2^<0.01 | -0.02  [-0.06, 0.01]  *f*^2^<0.01 | -0.004  [-0.01, 0.00]  *f*^2^<0.01 |
|  |  |  |  |  |

*Note*. Results are unstandardized regression coefficients, asterisks to indicate significance, [95% confidence intervals], Cohen’s *f*^2^. ** p* < .05. TST = Total Sleep Time; SOL = Sleep Onset Latency (square-root transformed); WASO = Wake After Sleep Onset (square-root transformed), SE = Sleep Efficiency (winzorised). Adjusted models included baseline and daily covariates: age; sex; race/ethnicity; body mass index; employment status; English language acculturation; subjective social status; time spent in Melbourne; COVID-19 period; student status; smoking status; alcohol consumption; day of week; composite phase deviation.

Table S4. Covariates as Predictors of EEG-assessed Sleep

|  | **TST** | **SOL** | **WASO** | **SE** | **SWS** | **REM** |
| --- | --- | --- | --- | --- | --- | --- |
| **Lagged sleep** | -0.15** | -0.05 | -0.02 | -0.11** | 0.01 | -0.12** |
| **Age** | -0.85 | -0.04 | 0.09 | -0.22 | -0.02 | -2.32 |
| **Sex** | 12.29 | 0.05 | 0.30 | -0.96 | 8.26 | 17.98 |
| **Race (Ref: Asian)** |  |  |  |  |  |  |
| **Race (Other)** | -26.88 | 0.40 | 1.14 | -5.01 | 4.19 | -19.45 |
| **Race (White/European)** | 16.19 | -0.32 | 0.11 | 1.35 | -9.01 | 18.75 |
| **Employment** | 15.42 | 0.42 | 0.68 | -1.50 | 3.55 | -3.05 |
| **Student status** | 19.88 | -0.10 | -0.11 | 0.40 | 4.39 | -2.85 |
| **Time in Victoria** | -0.55 | 0.02 | -0.12 | 0.08 | -1.78 | 2.65 |
| **Eng Acculturation** | -11.85 | 0.03 | 0.16 | -0.78 | 1.29 | -0.14 |
| **Subjective Social Status** | 8.53 | -0.16 | 0.06 | 0.52 | 0.45 | -0.40 |
| **COVID period** | 10.77 | -0.06 | 0.06 | 0.10 | -14.41** | 8.19 |
| **BMI** | 1.39 | 0.02 | -0.03 | 0.15 | 0.56 | 0.17 |
| **Smoking Status** | 10.63 | 1.02 | -0.21 | -1.20 | -10.67 | 11.55 |
| **Alcohol (Ref: Abstainer)** |  |  |  |  |  |  |
| **Alcohol (at risk)** | -4.56 | -0.93 | -0.89 | 3.78 | 3.96 | 19.07 |
| **Alcohol (Moderate)** | -17.65 | -0.49 | -0.57 | 2.08 | -2.92 | 4.32 |
| **Composite Phase Deviation** | -16.36*** | -0.05 | 0.12 | 1.23*** | -2.81* | -5.41*** |
| **Day of Week (Ref: Monday)** |  |  |  |  |  |  |
| **Tuesday** | 19.36* | -0.67** | -0.15 | 2.99** | 0.28 | 5.12 |
| **Wednesday** | 14.91 | -0.56* | -0.12 | 2.37* | 4.06 | 4.18 |
| **Thursday** | 23.51* | -0.50* | -0.09 | 2.75** | 3.17 | 7.02 |
| **Friday** | 27.35** | -0.60* | -0.16 | 3.18*** | 2.39 | 9.25* |
| **Saturday** | 39.16*** | -0.47* | 0.36 | 1.97* | 3.05 | 10.69* |
| **Sunday** | 29.62** | -0.57* | 0.40 | 1.88* | 0.30 | 8.08* |

*Note.* Results presented are unstandardized coefficients. ** p* < .05. *** p* < .01. **** p* < .001. TST = Total Sleep Time; SOL = Sleep Onset Latency (square-root transformed); WASO = Wake After Sleep Onset (square-root transformed), SE = Sleep Efficiency (winzorised). BMI = Body Mass Index.

Table S5. Covariates as Predictors of Next-Day Stress

|  | **TST** | **SOL** | **WASO** | **SE** | **SWS** | **REM** |
| --- | --- | --- | --- | --- | --- | --- |
| **Lagged Stress** | 0.21*** | 0.21*** | 0.21*** | 0.21*** | 0.22*** | 0.20*** |
| **Age** | -0.16 | -0.19 | -0.20 | -0.20 | -0.18 | -0.20 |
| **Sex** | -1.15* | -1.05* | -1.08* | -1.10* | -1.05* | -0.95 |
| **Race (Ref: Asian)** |  |  |  |  |  |  |
| **Race (Other)** | 0.68 | 0.53 | 0.43 | 0.48 | 0.69 | 0.45 |
| **Race (White/European)** | -0.08 | 0.03 | -0.06 | -0.02 | -0.12 | 0.07 |
| **Employment** | 0.33 | 0.27 | 0.37 | 0.39 | 0.51 | 0.41 |
| **Student status** | 0.10 | 0.03 | -0.02 | -0.004 | 0.10 | -0.002 |
| **Time in Victoria** | 0.29 | 0.29 | 0.28 | 0.27 | 0.24 | 0.30 |
| **Eng Acculturation** | 0.49* | 0.41 | 0.41 | 0.42 | 0.45* | 0.44 |
| **Subjective Social Status** | -0.23 | -0.17 | -0.21 | -0.19 | -0.19 | -0.20 |
| **COVID period** | 0.55 | 0.58 | 0.62 | 0.59 | 0.28 | 0.64 |
| **BMI** | -0.10 | -0.10 | -0.09 | -0.09 | -0.09 | -0.10 |
| **Smoking Status** | -0.32 | -0.28 | -0.21 | -0.15 | -0.30 | -0.13 |
| **Alcohol (Ref: Abstainer)** |  |  |  |  |  |  |
| **Alcohol (at risk)** | -0.77 | -0.44 | -0.63 | -0.61 | -0.70 | -0.58 |
| **Alcohol (Moderate)** | -0.19 | -0.07 | -0.18 | -0.18 | -0.25 | -0.19 |
| **Composite Phase Deviation** | -0.04 | -0.004 | -0.01 | -0.03 | -0.02 | -0.03 |
| **Day of Week (Ref: Monday)** |  |  |  |  |  |  |
| **Tuesday** | 0.08 | 0.06 | 0.02 | 0.07 | 0.04 | 0.05 |
| **Wednesday** | 0.23 | 0.21 | 0.19 | 0.22 | 0.22 | 0.21 |
| **Thursday** | 0.19 | 0.13 | 0.11 | 0.15 | 0.15 | 0.15 |
| **Friday** | -0.20 | -0.25 | -0.27 | -0.23 | -0.26 | -0.23 |
| **Saturday** | 0.16 | 0.09 | 0.08 | 0.10 | 0.10 | 0.12 |
| **Sunday** | 0.43* | 0.37 | 0.35 | 0.37 | 0.36 | 0.38 |

*Note.* Results presented are unstandardized coefficients. ** p* < .05. **** p* < .001. TST = Total Sleep Time; SOL = Sleep Onset Latency (square-root transformed); WASO = Wake After Sleep Onset (square-root transformed), SE = Sleep Efficiency (winzorised). BMI = Body Mass Index. All sleep variables are EEG-assessed.
